# Age at first birth and cardiovascular risk factors in the 1958 British birth cohort

**DOI:** 10.1136/jech-2016-208196

**Published:** 2017-03-07

**Authors:** Rebecca E Lacey, Meena Kumari, Amanda Sacker, Anne McMunn

**Affiliations:** 1 Department of Epidemiology and Public Health, University College London, London, UK; 2 Institute for Social and Economic Research, University of Essex, Colchester, UK

**Keywords:** Cardiovascular disease, Life course epidemiology, Cohort studies, DEMOGRAPHY, MATERNAL HEALTH

## Abstract

**Background:**

To assess relationships between age at first birth and cardiovascular risk factors in a large longitudinal study of men and women. By assessing associations for both genders, we were able to investigate biological versus social and behavioural explanations from early life through to adulthood.

**Methods:**

Multiply-imputed data on more than 7600 men and women of a British birth cohort study (National Child Development Study, 1958 British birth cohort) were used. Cardiovascular risk factors at age 44/45 years included body mass index, waist:hip ratio, blood pressure (systolic and diastolic), cholesterol (total, low and high-density lipoprotein), triglycerides, glycated haemoglobin, C reactive protein, von Willebrand factor and fibrinogen. Age at first birth was categorised as <20 years, 20–24 years, 25–29 years, 30–34 years or >34 years.

**Results:**

Being younger than 20 years of age at time of first birth was associated with an adverse cardiovascular profile by mid-life. Conversely, older parents had a lower cardiovascular risk as captured by lower body mass index, waist:hip ratio, blood pressure, high and low-density lipoprotein cholesterol, triglycerides, glycated haemoglobin, C reactive protein and fibrinogen. The relationship between age at first birth and cardiovascular risk factors was graded. Few differences between men and women were observed. Associations were largely unchanged after adjustment for early life factors but were partially mediated through adult social and behavioural factors.

**Conclusions:**

Age at first birth is inversely associated with differences in cardiovascular risk factors in mid-life in a large prospective birth cohort. Our results potentially suggest a social and behavioural rather than a biological explanation.

## Introduction

Age at first birth may have long-term implications for the health of men and women. For instance, a young age at first birth (<20 years) has previously been linked to increased risk of cardiovascular disease and mortality in American women.^1–4^ Similarly, Finnish women who had their first child before age 25 years had increased risk of myocardial infarction and arrhythmia.^5^ In contrast, the biodevelopmental view suggests that childbearing should occur earlier in life, shortly after the reproductive system has matured,^1^ although this theory has received little support from empirical population studies. Most previous research has focused on the health of women; however, more recently, work on age at first birth and later health has been extended to look at the long-term health of young fathers. A recent study showed that young fatherhood was associated with higher mortality in Finland largely due to cardiovascular disease and higher allostatic load in England.^6^ ^7^ Research comparing men and women is useful for unpicking biological versus other explanations. Hardy *et al*^8^ compared the age at first birth of men and women in the Medical Research Council (MRC) National Survey of Health and Development (NSHD, the 1946 British birth cohort) in relation to multiple cardiovascular risk factors and found that an early age of first birth was associated with raised blood pressure (BP), body mass index (BMI) and waist:hip ratios of both men and women, suggesting that the mechanisms involved may be more social and behavioural than biological. However, the limitation of using the NSHD is that there is little variation in the timing of first birth, particularly among women.

There is a plausible biological pathway between early pregnancy and increased risk of cardiovascular disease. Women who experience pregnancy prior to age 20 years are more likely to develop eclampsia, pregnancy-related hypertension, lasting insulin resistance and altered cholesterol profiles.^9^ ^10^ There is some suggestion that these pregnancy-related changes might exert a greater influence on cardiovascular disease development, the earlier they occur, operating partially through increasing parity.^11^ An alternative to the biological explanation for associations between age at first birth and later health is that age at first birth is correlated with socioeconomic position, as well as various behavioural factors, which are likely to apply to both men and women. Later parenthood may be associated with greater social control of health behaviours that influence cardiovascular risk 12, such as smoking and alcohol consumption. Also, early parenthood may result in reduced opportunities, financial resources and disruption of educational and career trajectories^13–15^, resulting in stress and consequently poorer health. In addition, there is the potential that early life circumstances, such as social disadvantage, pubertal timing and health, may confound associations between age at first birth and later health. For instance, social disadvantage, young age at puberty and parental separation are all known to increase the likelihood of early parenthood and worse health in adulthood.^16^ Thus far, relatively little is known about why age at first birth is associated with later health.

The aim of the present study was to extend the work of Hardy *et al*^8^ to investigate the relationship between age at first birth and cardiovascular risk factors in mid-life in a larger birth cohort of British men and women which had greater variation in age at first birth and a larger number of cardiovascular risk factors available. By comparing men and women, we were able to evaluate the extent to which a biological mechanism might be at play or whether social and behavioural mechanisms are a more likely explanation. In particular, we assessed whether associations between age at first birth and cardiovascular risk factors were explained by differences in early life factors (eg, childhood socioeconomic position) or by adult social and behavioural factors.

## Methods

This study used the National Child Development Study (NCDS) (the 1958 British birth cohort study). This multidisciplinary study recruited 17 415 babies born in one week of 1958 (98.2% of total births that week) in Great Britain.^17^ Information on health, economic, social and developmental factors has been collected from participants at ages 7, 11, 16, 23, 33, 42, 44/45, 46, 50 and 55 years. Ethical approval for the study was obtained from the multicentre research ethics committee and informed consent was obtained from all participants.^18^ The survey at age 44/45 years was a biomedical survey on a subsample of participants (n=9377, 77.9% of those targeted),^19^ during which blood samples were collected. This study therefore used data up to age 44/45 years when the assessment of cardiovascular risk factors was possible.

### Cardiovascular risk factors

Anthropometric and blood pressure measures, and blood samples, were obtained by a trained study nurse. These included standing height (cm), weight (kg), waist (cm) and hip circumference (cm). From these, body mass index (BMI) and waist:hip ratios were calculated. Three blood pressure measurements were taken and the third systolic and diastolic measurements were used in this study. Participants taking medications affecting blood pressure had their systolic blood pressure increased by 10 mm Hg and diastolic by 5 mm Hg as recommended.^20^ These medications included β-blockers, drugs affecting the renin–angiotensin system, calcium channel blockers and diuretics. Total cholesterol, triglycerides and high-density lipoprotein (HDL) cholesterol were measured from non-fasting blood samples. Low density lipoprotein (LDL) cholesterol was calculated from trigylcerides and HDL cholesterol values using the Friedewald formula. Glycated haemoglobin (HbA1c) was measured on citrated whole blood by ion exchange high-performance liquid chromatography.^19^ Trigylcerides and HbA1c values were log-transformed to reduce positive skew. Three inflammatory markers were also measured from blood samples—fibrinogen, C reactive protein (CRP) and von Willebrand factor (vWF). Values of all three were positively skewed and hence log-transformed for analyses. Participants with CRP values ≥10 mg/L (n=184), indicative of recent pathology or trauma,^21^ were excluded from analyses.

### Age at first birth

Detailed fertility histories were collected at each adult survey. This information was used to derive the age of the participant at the time of first birth prior to age 44 years (outcome assessment). This was categorised into the following age groups: ≤20, 20–24, 25–29, 30–34 and >34 years. A continuous version of this variable was also used (range 13–43 years).

### Early life factors

Early life factors known to be associated with both age at first birth and later health were included in the analyses. Father's social class (Registrar General's Social Class schema (RGSC): professional (I), managerial and technical (II), skilled non-manual (IIINM), skilled manual (IIIM), semi-killed manual (IV) and unskilled (V)) at age 11 years was used as a measure of childhood socioeconomic position, as were whether the participants' parents had remained in full-time education beyond minimum school leaving age (asked at birth for the mother and age 7 years for the father). Financial hardship at age 11 years and whether the cohort member had experienced parental separation during childhood were included. The Rutter behaviour scale A (mother-reported) was included as an indicator of emotional health at age 11 years. Finally, a doctor-rated indicator of pubertal development at age 11 years was included as early age at puberty has previously been linked to early sexual activity^22^ and also cardiovascular risk factors.^23^ This was derived separately for boys and girls, and combined two aspects of pubertal development: growth of pubic hair (boys and girls), genital development (boys only) and breast development (girls only). High scores on this variable reflect more advanced pubertal development.

### Adult behavioural and social factors

**Figure 1:**
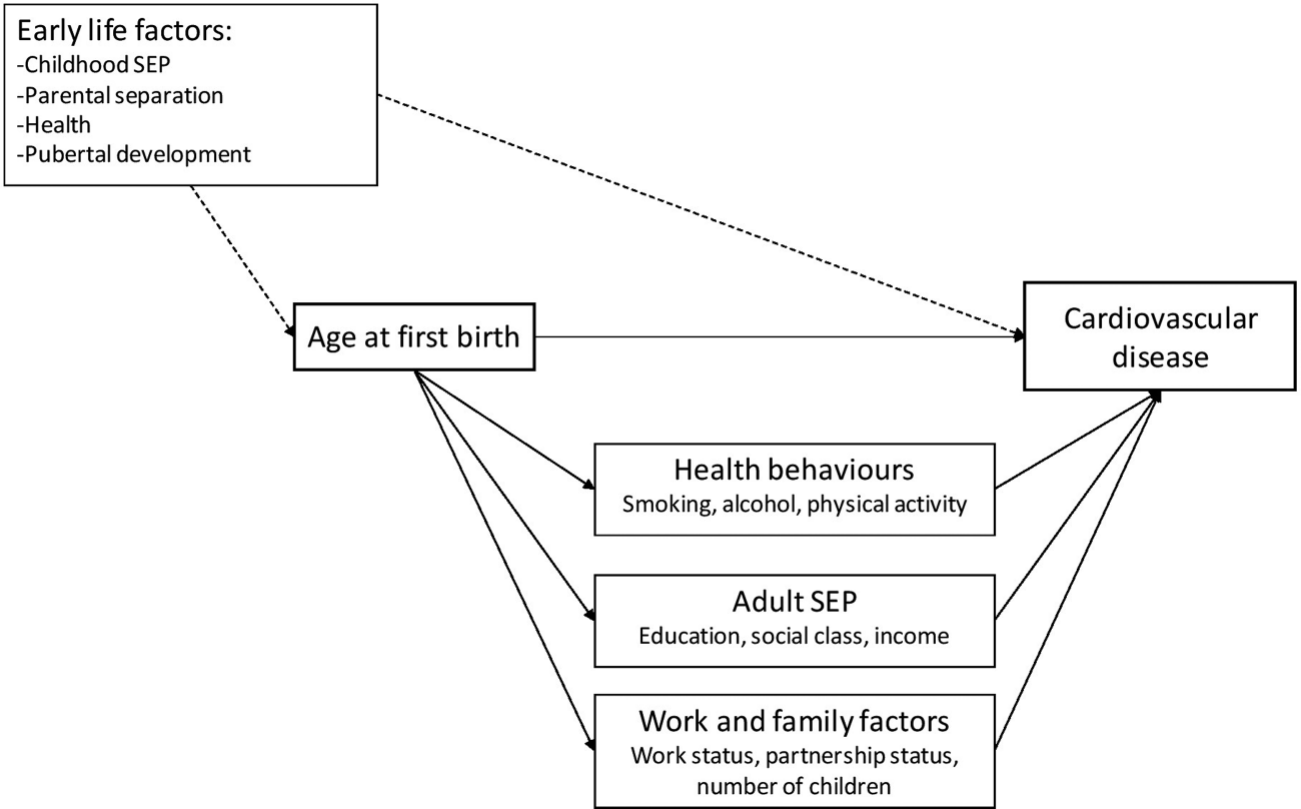
Conceptual diagram showing the relationship between age at first birth and cardiovascular disease, showing the role of early life factors and social and behavioural mediators. SEP, socioeconomic position.

Adult behavioural factors included were smoking status at age 42 years (never smoked, ex-smoker or current smoker), participation in regular physical activity and harmful drinking (AUDIT questionnaire:^24^ a score of 8 or more was indicative of harmful drinking). Adult social factors included measures of socioeconomic position. Educational attainment was derived as the highest qualification achieved by age 23 years and categorised as: no qualifications, Certificate of Secondary Education (CSE) or Ordinary-level (O-level), Advanced-level (A-level) or higher qualification/degree. The highest occupational social class in the household was derived at age 42 years (RGSC) and the same categories used as above. Housing tenure at age 42 years was categorised as owned outright or with a mortgage, privately-rented, social housing and other. Gross income in quintiles was derived at age 46 years, assuming that there would be little change in income quintiles during the year beyond outcome measurement. Working status at age 42 years was defined as employed full-time, employed part-time, unemployed, sick or disabled, looking after the home and family and other (including those retired, in education or other). Participants reported their partnership status at age 42 years as married, cohabiting, single (never married) or separated/divorced/widowed. The number of biological children the cohort member had had by age 44 years was also reported (range zero to 10). This variable was used to restrict the analyses to those who had had children by age 44 years. Figure 1 depicts the mediators and early life factors of interest in this study.

### Statistical analyses

#### Missing data

Supplementary table 1 compares the distribution of all analysis variables between those with completely observed data and those with missing data. The table shows that a complete case analysis would be inappropriate in this case as, consistent with many longitudinal studies, those with complete data were more socially advantaged than those with missing data (ie, data were not missing completely at random).^25^ Missing information was accounted for using multiple imputation, assuming that data were missing at random. Twenty imputed data sets were created using multiple imputation by chained equations. The imputation model included all analysis variables plus auxiliary variables from previous and subsequent waves, such as repeated measures of social class. The approach of multiple imputation followed by deletion^26^ was followed, imputing missing information for all participants but then excluding those with missing data on each outcome of interest. The sample for each cardiovascular risk factor outcome therefore varies (see ns reported in table 1). The numbers of men and women with each observed outcome are reported in table 1. Descriptive analyses are presented for those participants who had at least one child and at least one observed outcome (n=7682).

**Table 1:** Characteristics of men and women in the NCDS study sample

	Women		Men		
	N (%)	Mean (SD)	N (%)	Mean (SD)	p Gender difference
Cardiovascular risk factors					
BMI (kg/m^2^)	3885	26.97 (5.45)	3677	27.87 (4.12)	<0.001
Waist:hip ratio	3922	0.81 (0.06)	3715	0.93 (0.06)	<0.001
Systolic BP (mm Hg)	3896	119.40 (16.30)	3706	131.80 (15.29)	<0.001
Diastolic BP (mm Hg)	3896	74.82 (10.73)	3706	81.33 (10.88)	<0.001
Total cholesterol (mmol/L)	3286	5.69 (0.99)	3204	6.06 (1.14)	<0.001
LDL cholesterol (mmol/L)	3222	3.28 (0.86)	2920	3.56 (0.93)	<0.001
HDL cholesterol (mmol/L)	3283	1.68 (0.40)	3192	1.43 (0.33)	<0.001
Triglycerides (mmol/L)*	3279	1.30 (0.90, 2.00)	3192	2.10 (1.40, 3.00)	<0.001
HbA1c (%)*	3332	5.10 (4.90, 5.30)	3243	5.20 (5.00, 5.50)	<0.001
C reactive protein (mg/L)*	3145	0.94 (0.49, 1.90)	3095	0.93 (0.42, 2.20)	0.091
Fibrinogen (g/L)*	3245	2.95 (2.59, 3.38)	3140	2.82 (2.48, 3.20)	<0.001
vWF*	3244	115 (91, 144)	3151	120 (94, 148)	<0.001
Age at first birth (years)†					
<20	546 (13.8)				<0.001
20–24	1420 (36.0)				
25–29	1153 (29.1)		1268 (34.1)		
30–34	496 (12.6)		744 (19.7)		
>34	337 (8.5)		609 (16.1)		
Mean (SD)		25.37 (5.68)		28.15 (5.89)	

*Median and IQR presented as measure is positively skewed.

†Age at first birth %s reported for those with at least one observed outcome (n=7682), n reported from first imputed data set as an indication of the number of participants in each group (the number will vary slightly across imputed data sets).

BMI, body mass index; BP, blood pressure; HbA1c, glycated haemoglobin; HDL, high density lipoprotein; LDL, low density lipoprotein; NCDS, National Child Development Study; vWF, von Willebrand Factor.

10.1136/jech-2016-208196.supp1 supplementary table Distribution of all analysis variables for complete cases, those with some missing data and those with imputed data

#### Regression analyses

Associations between age at first birth and cardiovascular risk factors were tested using linear regression (reference group: age 20–24 years, modal category for whole sample). All analyses were stratified by gender. First, the crude association between age at first birth and each outcome was estimated. Second, early life factors were included in the model. Finally, adult lifestyle and behavioural factors, and the number of children were additionally included in the final model. Wald tests were conducted throughout to test for possible gender-age at first birth interactions. Tests for trend were conducted throughout to highlight the trend in association between age at first birth and each outcome. In models where the outcome variable was log-transformed (triglycerides, HbA1c, CRP, fibrinogen and vWF), regression coefficients have been converted to percentage difference to aid interpretation.

Sensitivity analyses were conducted excluding those taking lipid-lowering medications (for trigylcerides and cholesterol regressions), steroids (for CRP regressions) and antidiabetes medications (for HbA1c regressions). An additional sensitivity analysis was conducted for women by additionally including menopause status (premenopause/postmenopause/hormone replacement therapy/hysterectomy) in a final model. We also conducted sensitivity analyses including socioeconomic variables, such as housing tenure, social class and work status, from earlier ages (16, 23 and 33) to assess whether timing of childbearing is a consequence of earlier socioeconomic factors. None of these sensitivity analyses altered the results. All analyses were conducted using Stata V.13.( StataCorp. Stata version 14.2. 2015)

## Results

The characteristics of men and women in the study sample with respect to age at first birth and cardiovascular risk factors are shown in table 1. Sex differences in all cardiovascular risk factors were found (higher in men), with the exception of CRP and fibrinogen. Women in this cohort tended to be younger than men at time of first birth. With the exception of HDL cholesterol which increased with older age at first birth, all outcomes showed a decreasing trend by increasing age at first birth for women (table 2). For men, there was no statistical trend in total or LDL cholesterol values by age at first birth.

**Table 2:** Unadjusted means of cardiovascular risk factors at age 44/45 years by age at first birth in the NCDS

	Age at first birth					
	<20 years	20–24 years	25–29 years	30–34 years	>34 years	
	Mean (95% CI)	Mean (95% CI)	Mean (95% CI)	Mean (95% CI)	Mean (95% CI)	p Trend
BMI						
Men	29.45 (28.65 to 30.24)	28.22 (27.95 to 28.49)	27.83 (27.61 to 28.06)	27.66 (27.37 to 27.96)	27.24 (26.88 to 27.60)	<0.001
Women	27.96 (27.44 to 28.49)	27.61 (27.32 to 27.90)	26.51 (26.21 to 26.81)	25.89 (25.44 to 26.33)	25.58 (25.02 to 26.14)	<0.001
Waist:hip ratio						
Men	0.95 (0.94 to 0.96)	0.94 (0.93 to 0.94)	0.93 (0.92 to 0.93)	0.93 (0.92 to 0.93)	0.92 (0.91 to 0.92)	<0.001
Women	0.83 (0.82 to 0.83)	0.82 (0.81 to 0.82)	0.81 (0.80 to 0.81)	0.80 (0.80 to 0.81)	0.80 (0.79 to 0.81)	<0.001
SBP						
Men	135.35 (132.62 to 138.07)	132.67 (131.64 to 133.69)	132.09 (131.23 to 132.95)	131.80 (130.69 to 132.92)	128.89 (127.71 to 130.06)	<0.001
Women	121.50 (120.01 to 122.99)	120.08 (119.20 to 120.97)	118.88 (117.94 to 119.82)	118.13 (116.77 to 119.48)	116.38 (114.69 to 118.08)	<0.001
DBP						
Men	84.07 (82.19 to 85.94)	81.92 (81.20 to 82.63)	81.55 (80.95 to 82.15)	81.58 (80.75 to 82.40)	79.00 (78.14 to 79.87)	<0.001
Women	76.24 (75.29 to 77.19)	75.13 (74.55 to 75.70)	74.42 (73.79 to 75.05)	74.37 (73.44 to 75.31)	73.06 (71.94 to 74.17)	<0.001
Total cholesterol						
Men	6.11 (5.90 to 6.32)	6.09 (6.01 to 6.18)	6.06 (6.00 to 6.13)	6.08 (5.98 to 6.17)	5.99 (5.90 to 6.09)	0.163
Women	5.81 (5.71 to 5.76)	5.70 (5.64 to 5.76)	5.65 (5.59 to 5.72)	5.60 (5.51 to 5.70)	5.62 (5.50 to 5.74)	0.003
LDL cholesterol						
Men	3.68 (3.49 to 3.87)	3.56 (3.49 to 3.63)	3.55 (3.49 to 3.61)	3.58 (3.50 to 3.66)	3.54 (3.46 to 3.61)	0.459
Women	3.36 (3.28 to 3.44)	3.32 (3.28 to 3.37)	3.27 (3.21 to 3.33)	3.16 (3.07 to 3.24)	3.18 (3.08 to 3.29)	<0.001
HDL cholesterol						
Men	1.37 (1.31 to 1.42)	1.41 (1.39 to 1.44)	1.44 (1.42 to 1.46)	1.44 (1.41 to 1.47)	1.44 (1.41 to 1.47)	0.042
Women	1.60 (1.57 to 1.64)	1.64 (1.62 to 1.66)	1.70 (1.67 to 1.73)	1.77 (1.73 to 1.81)	1.82 (1.76 to 1.87)	<0.001
Triglycerides*						
Men	2.30 (1.50 to 2.80)	2.10 (1.40 to 3.10)	2.10 (1.40 to 3.00)	2.00 (1.40 to 3.10)	2.00 (1.30 to 2.80)	0.005
Women	1.50 (1.00 to 2.20)	1.40 (1.00 to 2.00)	1.30 (0.90 to 1.90)	1.20 (0.90 to 1.70)	1.20 (0.80 to 1.70)	<0.001
HbA1c*						
Men	5.20 (5.00 to 5.50)	5.30 (5.00 to 5.50)	5.20 (5.00 to 5.50)	5.20 (5.00 to 5.50)	5.20 (5.00 to 5.40)	<0.001
Women	5.20 (5.00 to 5.40)	5.10 (4.90 to 5.40)	5.10 (4.90 to 5.30)	5.10 (4.90 to 5.30)	5.10 (4.90 to 5.30)	<0.001
CRP*						
Men	1.32 (0.72 to 2.56)	1.09 (0.57 to 2.17)	0.92 (0.47 to 1.79)	0.84 (0.44 to 1.78)	0.79 (0.44 to 1.55)	<0.001
Women	1.33 (0.56 to 2.79)	1.00 (0.47 to 2.41)	0.84 (0.38 to 2.00)	0.84 (0.38 to 1.96)	0.73 (0.32 to 1.66)	<0.001
Fibrinogen*						
Men	2.95 (2.57 to 3.36)	2.90 (2.63 to 3.45)	2.82 (2.47 to 3.16)	2.76 (2.44 to 3.16)	2.71 (2.41 to 3.12)	<0.001
Women	3.09 (2.70 to 3.54)	3.01 (2.63 to 3.45)	2.93 (2.58 to 3.31)	2.85 (2.51 to 3.22)	2.78 (2.45 to 3.17)	<0.001
vWF*						
Men	124 (94 to 149)	122 (99 to 147)	122 (95 to 151)	113 (88 to 144)	118 (94 to 148)	0.031
Women	116 (94 to 144)	118 (93 to 148)	114 (91 to 144)	109 (87 to 138)	106 (87 to 136)	<0.001

*Median (IQR) presented as outcome is positively skewed.

BMI, body mass index; CRP, C reactive protein; DBP, diastolic blood pressure; HbA1c, glycated haemoglobin; HDL, high-density lipoprotein; LDL, low-density lipoprotein; NCDS, National Child Development Study; SBP, systolic blood pressure; vWF, von Willebrand Factor.

Table 3 shows the crude (unadjusted) associations between age at first birth and cardiovascular risk factors. With the exception of HDL cholesterol and BMI, none of the associations differed for men and women. Overall increasing age at first birth was inversely associated with cardiovascular risk factors. Compared to men and women who had their first birth at age 20–24 years, those who were <20 years had higher BMIs by age 44/45 years, and those who were older than 25 years had lower BMIs. A similar pattern of results was seen for waist:hip ratios, SBP, DBP, triglycerides, HbA1c, CRP and fibrinogen. Although not statistically different, the relationship between age at first birth and BP (SBP and DBP) appeared to be more pronounced for men. Also, the association between age at first birth and vWF was more pronounced for women (test for trend: p<0.001). There was no relationship between age at first birth and LDL or total cholesterol in this study; therefore, these outcomes were not considered in further regression analyses.

**Table 3:** Unadjusted regression coefficients (95% CIs) for crude association between age at first birth and cardiovascular risk factors at age 44/45 years in the NCDS

	<20 years	20–24 years	25–29 years	30–34 years	>34 years	p Gender difference	p Trend
BMI							
Men	1.23 (0.46 to 1.99)	Ref	−0.38 (−0.74 to −0.03)	−0.56 (−0.97 to −0.14)	−0.98 (−1.42 to −0.54)	0.005	<0.001
Women	0.35 (−0.19 to 0.90)	Ref	−1.10 (−1.54 to −0.67)	−1.73 (−2.29 to −1.17)	−2.03 (−2.68 to −1.38)		<0.001
Waist:hip ratio							
Men	0.01 (0.002 to 0.02)	Ref	−0.01 (−0.01 to −0.003)	−0.01 (−0.02 to −0.005)	0.02 (−0.03 to −0.01)	0.895	<0.001
Women	0.01 (0.002 to 0.02)	Ref	−0.01 (−0.02 to −0.01)	−0.01 (−0.02 to −0.01)	−0.02 (−0.03 to −0.01)		<0.001
SBP							
Men	2.68 (−0.10 to 5.46)	Ref	−0.58 (−1.87 to 0.72)	−0.87 (−2.39 to 0.65)	−3.78 (−5.40 to −2.16)	0.826	<0.001
Women	1.42 (−0.24 to 3.07)	Ref	−1.20 (−2.50 to 0.10)	−1.96 (−3.69 to −0.22)	−3.70 (−5.67 to −1.73)		<0.001
DBP							
Men	2.15 (0.16 to 4.14)	Ref	−0.37 (−1.30 to 0.56)	−0.34 (−1.41 to 0.74)	−2.92 (−4.06 to −1.77)	0.572	<0.001
Women	1.11 (0.02 to 2.20)	Ref	−0.71 (−1.57 to 0.14)	−0.75 (−1.89 to 0.38)	−2.07 (−3.37 to −0.77)		<0.001
Total cholesterol							
Men	0.02 (−0.20 to 0.24)	Ref	−0.03 (−0.14 to 0.07)	−0.02 (−0.14 to 0.10)	−0.10 (−0.23 to 0.03)	0.738	0.163
Women	0.10 (−0.01 to 0.21)	Ref	−0.05 (−0.13 to 0.04)	−0.10 (−0.21 to 0.02)	−0.08 (−0.21 to 0.06)		0.003
LDL cholesterol							
Men	0.12 (−0.07 to 0.31)	Ref	−0.01 (−0.10 to 0.08)	0.02 (−0.09 to 0.12)	−0.03 (−0.13 to 0.08)	0.138	0.458
Women	0.03 (−0.06 to 0.13)	Ref	−0.06 (−0.13 to 0.02)	−0.17 (−0.26 to −0.07)	−0.14 (−0.26 to −0.02)		<0.001
HDL cholesterol							
Men	−0.05 (−0.11 to 0.02)	Ref	0.03 (−0.004 to 0.06)	0.03 (−0.01 to 0.06)	0.03 (−0.01 to 0.06)	<0.001	0.042
Women	−0.04 (−0.08 to 0.01)	Ref	0.06 (0.03 to 0.10)	0.13 (0.08 to 0.17)	0.18 (0.13 to 0.23)		<0.001
Triglycerides*							
Men	−0.95 (−11.15 to 10.43)	Ref	−2.22 (−7.23 to 3.07)	−4.26 (−9.88 to 1.71)	−7.93 (−13.55 to −1.94)	0.065	0.005
Women	10.77 (4.55 to 17.35)	Ref	−7.39 (−11.48 to −3.12)	−10.47 (−15.66 to −4.96)	−16.62 (−22.22 to −10.62)		<0.001
HbA1c*							
Men	−1.62 (−3.65 to 0.45)	Ref	−1.50 (−2.49 to −0.50)	−2.24 (−3.37 to −1.10)	−2.43 (−3.60 to −1.24)	0.264	<0.001
Women	1.21 (0.20 to 2.22)	Ref	−0.63 (−1.40 to 0.15)	−1.10 (−2.11 to −0.08)	−1.67 (−2.84 to −0.49)		<0.001
CRP*							
Men	19.34 (−1.75 to 44.96)	Ref	−16.41 (−23.85 to −8.25)	−20.57 (−28.65 to −11.58)	−22.71 (−30.89 to −13.57)	0.831	<0.001
Women	13.99 (0.03 to 29.90)	Ref	−20.69 (−28.37 to −12.19)	−19.42 (−29.49 to −7.91)	−29.99 (−39.95 to −18.37)		<0.001
Fibrinogen*							
Men	0.32 (−3.29 to 4.05)	Ref	−2.68 (−4.37 to −0.96)	−3.61 (−5.53 to −1.64)	−5.14 (−7.11 to −3.12)	0.172	<0.001
Women	2.18 (−0.10 to 4.51)	Ref	−2.47 (−4.17 to −0.74)	−5.04 (−7.22 to −2.80)	−7.85 (−10.30 to −5.34)		<0.001
vWF*							
Men	−1.28 (−7.37 to 5.20)	Ref	0.16 (−2.85 to 3.26)	−5.41 (−8.67 to −2.04)	−2.20 (−5.71 to 1.45)	0.365	0.031
Women	−1.41 (−4.94 to 2.25)	Ref	−3.03 (−5.75 to −0.22)	−7.19 (−10.62 to −3.63)	−7.16 (−11.12 to −3.03)		<0.001

*Regression results presented as % difference as outcomes were positively skewed and hence log-transformed for analyses.

BMI, body mass index; CRP, C reactive protein; DBP, diastolic blood pressure; HbA1c, glycated haemoglobin; HDL, high-density lipoprotein; LDL, low-density lipoprotein; NCDS, National Child Development Study; SBP, systolic blood pressure; vWF, von Willebrand Factor.

After accounting for differences in early life factors (table 4), such as parental separation, father's social class, puberty score, parental education, financial hardship and child behaviour, associations between age at first birth and cardiovascular risk factors largely remained unchanged, although an attenuation was observed for associations with BMI and BP, suggesting partial confounding by early life factors. The trend with vWF for men also became statistically non-significant after inclusion of early life factors. After including possible adult mediators (health behaviours, indicators of socioeconomic position, work and partnership status, and number of children), many of the associations between age at first birth and cardiovascular risk factors were attenuated further but remained statistically significant in most cases. A notable exception to this was the association between age at first birth and CRP which was no longer present for men or women in the fully adjusted models, although a statistically significant trend was still present for men. For both men and women, the attenuation was largely explained by differences in educational attainment (educational attainment increases with increasing age at first birth). Conversely, associations between age at first birth and BMI remained little changed after accounting for adult social and behavioural factors, and consequently differences by age at first birth were still seen for both men and women. This was also the case for BP for both men and women; for instance, men who had their first child prior to age 20 years had a higher DBP (2.02 mm Hg higher, 95% CI 0.04 to 4.00) and men aged >34 years at first birth had a lower DBP (2.55 mm Hg lower, 95% CI 3.75 to 1.36) compared to men who were 20–24 years. This same pattern was also apparent for SBP.

**Table 4:** Regression coefficients (95% CIs) for association between age at first birth and cardiovascular risk factors at age 44/45 years, accounting for early life and adult social and behavioural factors in the NCDS

	Adjusted for early life factors									Adjusted for adult social and behavioural factors				
	<20 years	20–24 years	25–29 years	30–34 years	>34 years	P gender difference	P trend	<20 years	20–24 years	25–29 years	30–34 years	>34 years	P gender difference	P trend
BMI														
Men	1.14 (0.37 to 1.90)	Ref	−0.24 (−0.60 to 0.12)	−0.37 (−0.78 to 0.05)	−0.70 (−1.14 to −0.25)	0.010	<0.001	1.08 (0.32 to 1.85)	Ref	−0.36 (−0.72 to 0.01)	−0.39 (−0.82 to 0.04)	−0.74 (−1.20 to −0.28)	0.023	<0.001
Women	0.19 (−0.34 to 0.73)	Ref	−0.82 (−1.26 to −0.39)	−1.36 (−1.91 to −0.80)	−1.64 (−2.28 to −0.99)		<0.001	−0.03 (−0.57 to 0.51)	Ref	−0.57 (−1.02 to −0.12)	−1.03 (−1.62 to −0.45)	1.41 (−2.08 to −0.74)		<0.001
Waist:hip ratio														
Men	0.01 (0.001 to 0.02)	Ref	−0.01 (−0.01 to −0.0002)	−0.01 (−0.01, −0.001)	−0.01 (−0.02 to −0.01)	0.933	<0.001	0.01 (−0.001 to 0.02)	Ref	−0.003 (−0.01 to 0.003)	−0.004 (−0.01 to 0.003)	−0.01 (−0.02 to −0.004)	0.746	<0.001
Women	0.01 (0.0004 to 0.01)	Ref	−0.01 (−0.01 to −0.002)	−0.01 (−0.02, −0.002)	−0.01 (−0.02 to −0.01)		<0.001	−0.0002 (−0.01 to 0.01)	Ref	−0.002 (−0.01 to 0.003)	−0.003 (−0.01 to 0.003)	−0.01 (−0.02 to −0.0001)		0.066
SBP														
Men	2.57 (−0.22 to 5.37)	Ref	−0.26 (−1.57 to 1.06)	−0.40 (−1.95, 1.16)	−3.16 (−4.81 to −1.52)	0.882	<0.001	2.48 (−0.29 to 5.26)	Ref	−0.21 (−1.56 to 1.14)	−0.22 (−1.82 to 1.39)	−2.99 (−4.68 to −1.29)	0.856	<0.001
Women	1.23 (−0.43 to 2.89)	Ref	−0.64 (−1.97 to 0.68)	−1.23 (−3.01, 0.54)	−2.94 (−4.95 to −0.94)		<0.001	1.07 (−0.62 to 2.76)	Ref	−0.60 (−1.97 to 0.76)	−1.30 (−3.15 to 0.54)	−3.08 (−5.18 to −0.98)		0.001
DBP														
Men	2.07 (0.08 to 4.07)	Ref	−0.08 (−1.03 to 0.86)	0.04 (−1.05, 1.14)	−2.40 (−3.57 to −1.22)	0.656	<0.001	2.02 (0.04 to 4.00)	Ref	−0.25 (−1.21 to 0.71)	−0.07 (−1.19 to 1.05)	−2.55 (−3.75 to −1.36)	0.781	<0.001
Women	1.02 (−0.07 to 2.11)	Ref	−0.31 (−1.18 to 0.56)	−0.25 (−1.41, 0.90)	−1.55 (−2.87 to −0.23)		0.002	0.95 (−0.16 to 2.06)	Ref	−0.30 (−1.19 to 0.60)	−0.27 (−1.48 to 0.93)	−1.69 (−3.08 to −0.31)		0.005
HDL cholesterol														
Men	−0.04 (−0.11 to 0.02)	Ref	0.02 (−0.01 to 0.05)	0.02 (−0.02 to 0.05)	0.01 (−0.03 to 0.05)	<0.001	0.241	−0.05 (−0.11 to 0.02)	Ref	0.01 (−0.02 to 0.04)	0.01 (−0.03 to 0.04)	0.01 (−0.03 to 0.05)	<0.001	0.241
Women	−0.02 (−0.07 to 0.02)	Ref	0.04 (0.001 to 0.07)	0.09 (0.04 to 0.14)	0.14 (0.09 to 0.19)		0.020	0.004 (−0.04 to 0.05)	Ref	0.01 (−0.03 to 0.04)	0.06 (0.01 to 0.10)	0.10 (0.04 to 0.15)		0.001
Triglycerides*														
Men	−1.49 (−11.67 to 9.85)	Ref	−1.48 (−6.58 to 3.91)	−3.44 (−9.19 to 2.68)	−6.74 (−12.56 to −0.53)	0.081	<0.001	−1.19 (−11.45 to 10.25)	Ref	−0.79 (−6.04 to 4.76)	−2.65 (−8.69v 3.77)	−5.56 (−11.68 to 0.97)	0.334	0.074
Women	9.76 (3.61 to 16.27)	Ref	−4.86 (−9.10 to −0.43)	−6.86 (−12.33 to −1.05)	−12.96 (−18.86 to −6.63)		<0.001	4.53 (−1.38 to 10.78)	Ref	−0.93 (−5.42 to 3.78)	−2.73 (−8.63 to 3.56)	−7.42 (−13.91 to −0.45)		0.011
HbA1c*														
Men	−1.91 (−3.93 to 0.15)	Ref	−1.28 (−3.72 to −0.27)	−2.07 (−3.22 to −0.92)	−2.15 (−3.34 to −0.92)	0.229	0.002	−1.95 (−3.95 to 0.10)	Ref	−0.53 (−1.55 to 0.49)	−1.24 (−2.42 to -0.05)	−1.68 (−2.91 to −0.43)	0.369	0.068
Women	1.05 (0.03 to 2.06)	Ref	−0.35 (−1.13 to 0.44)	−0.72 (−1.76 to 0.32)	−1.30 (−2.49 to −0.10)		0.001	0.40 (−0.61 to 1.42)	Ref	0.20 (−0.60 to 1.01)	−0.16 (−1.24 to 0.93)	−0.73 (−1.97 to 0.53)		0.337
CRP*														
Men	15.62 (−4.74 to 40.35)	Ref	−13.19 (−20.94 to −4.69)	−16.35 (−24.91 to −6.80)	−17.17 (−27.04 to −7.24)	0.754	<0.001	10.42 (−8.72 to 33.57)	Ref	−6.06 (−14.44 to 3.14)	−9.43 (−18.82 to 1.05)	−20.83 (−20.50 to 0.01)	0.698	0.045
Women	12.05 (−1.58 to 27.57)	Ref	−14.67 (−22.96 to −5.48)	−9.56 (−20.96, 3.49)	−21.06 (−32.36 to −7.89)		<0.001	3.84 (−8.95 to 18.42)	Ref	−8.83 (−17.90 to 1.24)	−1.85 (−14.68 to 12.92)	-14.82 (-27.46, 0.02)		0.089
Fibrinogen*														
Men	−0.18 (−3.76 to 3.53)	Ref	−2.05 (−3.77 to −0.31)	−2.79 (−4.75 to −0.79)	−4.10 (−6.13 to −2.03)	0.193	<0.001	−0.84 (−4.31 to 2.75)	Ref	−0.27 (−2.00 to 1.50)	−1.17 (−13.17 to 0.88)	−2.54 (−4.62 to −0.42)	0.490	0.073
Women	1.66 (−0.59 to 3.97)	Ref	−1.31 (−3.04 to 0.46)	−3.31 (−5.56 to −1.00)	−6.18 (−8.69 to −3.60)		<0.001	0.03 (−2.19 to 2.30)	Ref	0.05 (−1.73 to 1.86)	−1.92 (−4.27 to 0.48)	−4.75 (−7.37 to −2.05)		0.003
vWF*														
Men	−1.90 (−7.95 to 4.54)	Ref	1.01 (−1.82 to 4.41)	−3.92 (−7.27 to −0.45)	−0.31 (-3.96, 3.47)	0.376	0.352	−2.04 (−8.18 to 4.42)	Ref	2.21 (−0.95 to 5.47)	−2.98 (−6.49 to 0.65)	0.60 (−3.21 to 4.56)	0.435	0.728
Women	−1.58 (−5.12 to 2.09)	Ref	−2.12 (−4.91 to 0.76)	−5.95 (−9.49 to −2.26)	−5.83 (−9.90 to −1.56)		0.004	−2.55 (−6.12 to 1.12)	Ref	−1.19 (−4.09 to 1.81)	−4.84 (−8.59 to -0.93)	−4.75 (−9.06 to −0.23)		0.128

*Regression results presented as % difference as outcomes were positively skewed and hence log-transformed for analyses.

BMI, body mass index; CRP, C reactive protein; DBP, diastolic blood pressure; HbA1c, glycated haemoglobin; HDL, high-density lipoprotein; LDL, low-density lipoprotein; NCDS, National Child Development Study; SBP, systolic blood pressure; vWF, von Willebrand Factor.

Gender differences were apparent in the relationship between age at first birth and BMI. This appeared to be largely driven by the higher BMIs of young fathers (<20 years at first birth, men: 1.08, 95% CI 0.32 to 1.85, women: −0.03, 95% CI −0.57 to 0.51) and lower BMIs of older mothers (>34 years at first birth, women: −1.41, 95% CI −2.08 to −0.74, men: −0.74, 95% CI −1.20 to −0.28). A gender interaction was also present when HDL cholesterol was the outcome, with a stronger relationship seen for women. More specifically, HDL cholesterol levels increased with increasing age at becoming a mother.

## Discussion

Using data from a large British birth cohort of men and women, we found that age at first birth was linked to cardiovascular risk factors in mid-life. More specifically, increasing age at first birth was consistently associated with more favourable levels of our outcomes of interest (eg, decreased BMI, waist:hip ratio, BP, trigylcerides, HbA1c, CRP, fibrinogen and VWF, and increased HDL cholesterol). Conversely, becoming a parent prior to age 20 years was consistently associated with less favourable cardiovascular profiles, compared to becoming a parent between the ages of 20 and 24 years. This pattern of association was consistent across outcomes indicating anthropometric, cholesterol, lipid and inflammatory dimensions of cardiovascular risk. Associations between age at first birth and cardiovascular risk factors were largely similar for men and women, and remained on consideration of early life confounders, making a biological explanation unlikely. Consistent with this, there was evidence of partial mediation through adult social and behavioural factors. People who were younger at time of first birth in our study were more likely to be in disadvantaged socioeconomic positions, have lower educational attainment, riskier health behaviours (smoking, physically inactive and higher risk of problem alcohol consumption), less likely to be working, more likely to be cohabiting or separated from their partner and have more children than those who had children later. These factors in turn were associated with less favourable levels of cardiovascular risk factors. Some associations (eg, BMI, BP and fibrinogen for both men and women, and HDL cholesterol for women) remained after consideration of all these factors.

Our findings, taken together with the previous study by Hardy *et al*,^8^ which used an older cohort of British men and women (the MRC National Survey of Health and Development), suggest that there is reasonably strong evidence that the relationship between age at first birth and cardiovascular risk factors can largely be attributed to ‘social’ rather than ‘biological’ mechanisms. With the exception of HDL cholesterol in this study, associations between age at first birth and cardiovascular risk factors were similar for men and women, and were somewhat attenuated by inclusion of adult social and behavioural mediators. This was also the case in analyses of the English Longitudinal Study of Ageing which found that early parenthood was associated with increased allostatic load through wealth and health behaviours (smoking and physical activity).^7^ These three studies are the only studies which have compared relationships between age at first birth and objective health outcomes for both men and women. Additionally, only this and the NSHD study have been able to account for prospectively collected confounders and mediators. Our findings extend the work of Hardy and colleagues,^8^ suggesting that associations between age at first birth and health have persisted despite greater variation in age at first birth in this later cohort. An interesting extension to these two studies, using the 1946 and 1958 British birth cohorts, would be to replicate on the ‘next’ birth cohort, the 1970 British Cohort Study, where substantially more diversity in age at first birth and possibly associations with later health might be seen. In this study, we were also able to extend analyses to additionally investigate inflammatory markers which were not available in the 1946 British birth cohort at age 53 years. Our findings suggest that associations between age at first birth also relate to differences in inflammation (increasing age at first birth was associated with decreasing inflammation).

Previous studies which have analysed samples of men or women have shown that younger parents have worse health in terms of mortality,^1^ ^2^ ^6^ ^27^ heart disease^2^ ^28–30^ and cancer,^2^ and our study extends this work to objectively measured cardiovascular risk factors. Unlike our study, few of the previous studies have accounted for mediation through socioeconomic position, health behaviours and other adult factors, such as work and partnership status, instead often including either health behaviours or socioeconomic position. The most beneficial cardiovascular risk profiles in our study appeared to exist in men and women who had their first birth at >34 years. This is in contrast to other work which showed that American women had an increasing number of health problems if they had their first child beyond this age.^1^ ^31^ This difference could be due to the existence of an upper bound of this category in our study (maximum 43 years), but could also represent a true association between older parenthood and better health. Similar to previous work, the number of children did not fully explain relationships between age at first birth and cardiovascular risk factors in this study.^2^ ^8^ Associations between age at first birth and some outcomes remained even after adjustment for all variables of interest. This may be due to residual confounding, insufficient consideration of life course processes of social disadvantage or inclusion of mediators at only one point in the life course. It is possible that there were additional mediators which we were not able to include in this study; for instance, younger parents may have poorer health because of increased stigma and discrimination, and lower levels of social support.^32^ It is also possible that the mechanisms are different for men and women, in that a residual association could be explained by biological mechanisms for women but by another non-biological mechanism for men.

### Strengths and weaknesses of the study

First, non-fasting blood samples were collected in the biomedical survey. HDL cholesterol and HbA1c do not require fasting to be accurate and reliable; however, triglycerides are likely to be sensitive to fasting status.^33^ However, non-fasting triglycerides have previously been shown to be reliable markers of insulin resistance and risk factors for cardiovascular events, such as strokes, myocardial infarctions and cardiovascular mortality.^34^ Second, although we multiply-imputed missing information, it is possible that those who were not part of the biomedical survey were sicker than those included. Hence, the associations seen may have been underestimated in this sample. We also conducted multiple statistical tests in our study, meaning that there is a possibility that some associations found were due to chance. However, given that we found associations in the same direction across all outcomes, the message and conclusions of our study are likely to hold. Despite these limitations, this study also has a number of strengths. First, this dataset has detailed prospective information on early life factors, fertility histories and adult social and behavioural factors for both men and women. The availability of biomarkers allowed us to investigate objective markers of health in mid-life. The participants of the NCDS are broadly representative of men and women of a similar age who were born in Great Britain. Finally, we were able to extend Hardy *et al*'s^8^ study to a more recent, larger cohort of men and women with greater variation in age at first birth, more cardiovascular risk factor measures and outcomes measured at an earlier age.

In conclusion, this study suggests that a young age at first birth may be a risk factor for poorer cardiovascular health in later life, and that this is predominantly explained by differences in adult factors such as health behaviours, socioeconomic position and work and partnership status.

**What is already known on this subject?**

- Previous studies have found that early age at first birth is linked to poorer health; however, these have generally only investigated the health of women and used subjective health measures. Analysis of men and women in the 1946 British birth cohort found that a younger age at first birth was associated with cardiovascular risk; however, there was little variation in parental age in that cohort.

**What this study adds?**

- Using a larger British birth cohort with more variation in parental age (the 1958 British birth cohort, National Child Development Study), we find an improving cardiovascular profile with increasing age at first birth for both men and women. Our study included several objective cardiovascular risk factors in mid-life, including markers of inflammation, cholesterol, anthropometry, blood pressure and triglycerides. Social and behavioural, rather than biological, explanations appear to account for more of this association, although for many of our outcomes an association with age at first birth remained. Given the tendency towards later childbearing in the UK and other countries, this evidence is important in balancing the concerns in the medical community and the general public regarding the biological risks of later pregnancy.
