# Correction: *Age at first birth and cardiovascular risk factors in the 1958 british birth cohort*

**DOI:** 10.1136/jech-2016-208196corr1

**Published:** 2019-12-10

Lacey RE, Kumari M, Sacker A, *et al*. Age at first birth and cardiovascular risk factors in the 1958 British birth cohort. *J Epidemiol Community Health* 2017;71:691–8. doi: 10.1136/jech-2016-208196.

There is an error in table 1– part of the age at first birth information is missing for men.

The categories for men should be:

**Table:** 

Age at first birth (years)	N (%)
<20	147 (3.9)
20–24	962 (26.3)
25–29	1268 (34.1)
30–34	744 (19.7)
>34	609 (16.1)

