# Supplementary material for: Age at first birth and cardiovascular risk factors in the 1958 British birth cohort
Source: J Epidemiol Community Health. 2017 Mar 7;71(7):691–8. doi: 10.1136/jech-2016-208196 (PMC5485753; doi:10.1136/jech-2016-208196)
Supplement: supplementary table — Distribution of all analysis variables for complete cases, those with some missing data and those with imputed data [file jech-2016-208196supp001.pdf]

Supplementary table 1. Distribution of all analysis variables for complete cases, those with some missing data and those with imputed data

		Women			Men		
	Missing data[a] N (%)	Complete cases[b] Mean (SD)/ Median [IQR]	Missing data[c] Mean (SD)/ Median [IQR]	Imputed[d] Mean (SD)/ Median [IQR]	Complete cases[b] Mean (SD)/ Median [IQR]	Missing data[c] Mean (SD)/ Median [IQR]	Imputed[d] Mean (SD)/ Median [IQR]
BMI (kg/m^2)	119 (1.6)	26.47 (5.1)	27.05 (5.6)	26.97 (5.4)	27.82 (3.7)	27.82 (4.4)	27.87 (4.1)
Waist:hip ratio	45 (0.6)	0.81 (0.1)	0.81 (0.1)	0.81 (0.1)	0.93 (0.1)	0.93 (0.1)	0.93 (0.1)
SBP (mm Hg)	79 (1.1)	119.42 (15.9)	119.69 (16.3)	119.38 (16.3)	131.58 (15.1)	132.15 (15.5)	131.80 (15.3)
DBP (mm Hg)	81 (1.1)	74.81 (11.0)	75.08 (10.6)	74.80 (10.7)	81.58 (10.9)	81.56 (10.9)	81.32 (10.9)
Total cholesterol (mmol/L)	1211 (16.8)	5.68 (1.0)	5.70 (1.0)	5.69 (1.0)	6.08 (1.1)	6.07 (1.2)	6.06 (1.1)
LDL cholesterol (mmol/L)	1561 (21.6)	3.20 (1.0)	2.18 (2.2)	3.28 (0.9)	3.23 (1.5)	2.19 (2.4)	3.56 (0.9)
HDL cholesterol (mmol/L)	1226 (17.0)	1.73 (0.4)	0.97 (1.5)	1.68 (0.4)	1.43 (0.3)	0.81 (1.31)	1.43 (0.3)
Triglycerides (mmol/L)	1230 (17.0)	1.20 [0.9, 1.8]	1.30 [0.9, 2.0]	1.30 [0.9, 2.0]	2.10 [1.4, 3.0]	2.10 [1.4, 3.0]	2.10 [1.4, 3.0]
HbA1c (%)	1127 (15.6)	5.10 [4.9, 5.3]	5.10 [4.9, 5.3]	5.10 [4.9, 5.3]	5.20 [5.0, 5.4]	5.20 [5.0, 5.5]	5.20 [5.0, 5.5]
CRP (mg/L)	1312 (18.2)	0.94 [0.4, 2.2]	1.03 [0.4, 2.8]	0.93 [0.42, 2.20]	0.94 [0.5, 1.9]	0.96 [0.5, 2.1]	0.94 [0.49, 1.90]
Fibrinogen (g/L)	1321 (18.3)	2.92 [2.6, 3.3]	2.97 [2.6, 3.4]	2.95 [2.59, 3.38]	2.81 [2.5, 3.2]	2.83 [2.5, 3.2]	2.82 [2.48, 3.20]
vWF	1311 (18.2)	114 [90, 139]	116 [92, 146]	115 [91, 144]	118 [92, 146]	120 [95, 148]	120 [94, 148]

**Age at first birth**		%[e]	%	%	%	%	%
<20 years	539 (7.5)	9.2	18.5	13.8	2.6	6.1	3.9
20-24 years		34.6	37.2	36.0	23.5	31.5	26.3
25-29 years		33.5	25.4	29.1	37.9	31.0	34.1
30-34 years		13.7	11.1	12.6	20.2	17.5	19.7
>34 years		8.9	7.7	8.5	15.9	14.0	16.0
**Covariates**							
Early life factors							
Parental separation (0-16 yrs)	2221 (30.8)						
No		94.2	89.2	90.3	94.9	88.4	91.0
Yes		5.8	10.8	9.7	5.1	11.7	9.0
Puberty score (11 yrs)	1567 (21.7)	4 [2, 5]	4 [2, 5]	3 [2, 4]	3 [2, 4]	3 [2, 4]	4 [3, 5]
Financial hardship (11 yrs)	1292 (17.9)						
No		93.2	87.8	88.8	92.7	88.3	90.4
Yes		6.8	12.3	11.2	7.3	11.7	9.6
Rutter score (11 yrs)	1735 (24.0)	5 [3, 8]	6 [3, 8]	6 [3, 8]	6 [4, 8]	6 [4, 9]	6 [4, 9]
Father's social class (11 yrs)	1306 (18.1)						

I		6.1	5.0	5.1	8.4	5.6	6.7
II		21.0	18.6	19.4	20.7	17.1	18.5
IIINM		12.7	8.9	9.4	11.5	9.0	10.1
IIIM		41.8	42.7	42.4	40.8	43.3	41.5
IV		13.9	18.3	18.0	13.7	17.9	16.9
V		4.5	6.6	5.7	5.0	7.2	6.3
Mother's education (birth)	453 (6.3)						
Stayed beyond min age		25.9	25.1	26.1	28.0	24.5	27.2
Did not stay		74.1	74.9	73.9	72.0	75.5	72.8
Father's education (7 yrs)	1158 (16.0)						
Stayed beyond min age		27.7	23.2	24.8	27.4	22.4	24.5
Did not stay		72.3	76.9	75.2	72.6	77.6	75.5
Adult social and behavioural factors							
Educational attainment (23 yrs)	1287 (17.8)						
No qualifications		8.4	16.1	13.9	6.9	14.4	11.7
CSE/O-level		53.4	53.5	54.8	44.7	45.7	46.0
A-level		13.1	11.8	12.0	26.0	22.3	22.7
Higher/degree		25.1	18.5	19.3	22.4	17.7	19.6

Household social class (42 yrs)	710 (9.8)						
I		8.2	8.0	7.8	8.6	8.4	8.5
II		50.4	46.5	44.3	51.2	44.8	45.3
IIINM		24.4	21.8	21.4	18.9	19.1	19.2
IIIM		11.0	14.7	16.1	16.4	19.8	18.8
IV		5.3	7.4	8.5	4.3	6.0	6.1
V		0.8	1.6	1.9	0.6	1.9	2.1
Housing tenure (42 yrs)	253 (3.5)						
Own outright/mortgage		89.0	79.0	81.7	91.3	79.9	85.2
Privately-rented		2.3	4.0	3.1	2.7	5.1	3.6
Social housing		7.7	14.7	13.4	4.3	11.1	8.6
Other		1.0	2.4	1.8	1.7	3.9	2.6
Gross income quintiles (46 yrs)	2179 (30.2)						
Lowest quintile		20.0	28.1	26.8	11.3	21.1	17.6
2		21.0	20.0	20.5	19.6	19.6	19.1
3		19.9	18.2	18.7	19.7	19.7	20.4
4		18.3	17.5	17.3	23.2	18.4	20.0
Highest quintile		20.8	16.3	16.8	26.1	21.3	22.9

Working status (42 yrs)	231 (3.2)						
Employed full-time		47.5	44.1	39.6	96.3	86.7	90.6
Employed part-time		41.9	33.1	39.7	1.7	2.3	2.0
Unemployed		0.5	1.8	1.5	0.6	3.4	2.2
Sick or disabled		1.3	5.2	3.9	0.9	5.4	3.6
Looking after home/family		7.4	13.8	13.5	0.4	0.9	0.8
Retired/education/other		1.4	2.1	1.8	0.1	1.4	0.7
Partnership status (42 yrs)	254 (3.5)						
Married		81.5	69.4	76.5	83.0	68.3	80.4
Cohabiting		7.1	9.2	8.2	8.9	9.6	8.3
Single never married		1.5	8.4	2.6	0.9	12.1	2.6
Separated/divorced/widowed		10.0	13.0	12.7	7.1	10.0	8.7
Number of children (44 yrs)	423 (5.9)	2 [2, 3]	2 [1, 3]	2 [2, 3]	2 [2, 3]	2 [1, 2]	2 [2, 3]
Smoking status (42 yrs)	237 (3.3)						
Never smoked		47.0	45.2	45.8	48.6	42.8	43.9
Ex-smoker		28.1	23.9	25.2	28.1	25.6	27.2
Current smoker		24.9	30.9	29.0	23.3	31.6	28.9
AUDIT score (44 yrs)	577 (8.0)						

AUDIT score <8		86.3	83.8	84.4	63.2	62.6	63.2
AUDIT score>=8		13.7	16.2	15.6	36.8	37.4	36.8
Physical activity (42 yrs)	237 (3.3)						
No activity		23.3	27.8	27.0	20.8	25.3	23.4
Some activity		76.7	72.2	73.1	79.2	74.8	76.6
Menopause status (44 yrs) - women	108 (2.7)						
Pre-menopause		82.5	80.9	80.6			
Hysterectomy		6.4	5.9	6.4			
HRT		8.1	7.5	7.8			
Post-menopause		3.0	5.8	5.2			

a Presented for those cohort members who were parents and had at least one observed outcome (n=7222)

b Cohort members who had complete data on all analytic variables (n=1655)

c Cohort members who had missing information on at least one analytic variable (n=16903)

d Cohort members with imputed data (i.e. cohort members who were parents with at least one observed outcome - the analytic sample used in this study)

e %s reported as data are multiply imputed therefore numbers vary slightly across imputed datasets.

Abbreviations: A-level = Advanced level; AUDIT = Alcohol Use Disorders Identification Test; BMI = body mass index; CRP = C-reactive protein; CSE = Certificate of Secondary Education; DBP = diastolic blood pressure; HbA1c = glycated haemoglobin; HDL = high density lipoprotein; HRT = hormone

replacement therapy; IQR = interquartile range; LDL = low density lipoprotein; O-level = Ordinary level; SBP = systolic blood pressure; SD = standard deviation; vWF = von Willebrand Factor
